# A Mobile Application for Smart Computer-Aided Self-Administered Testing of Cognition, Speech, and Motor Impairment

**DOI:** 10.3390/s20113236

**Published:** 2020-06-06

**Authors:** Andrius Lauraitis, Rytis Maskeliūnas, Robertas Damaševičius, Tomas Krilavičius

**Affiliations:** 1 Department of Multimedia Engineering, Kaunas University of Technology, 50186 Kaunas, Lithuania; andrius.lauraitis@ktu.edu (A.L.); rytis.maskeliunas@ktu.lt (R.M.); 2 Department of Applied Informatics, Vytautas Magnus University, 44404 Kaunas, Lithuania; robertas.damasevicius@vdu.lt; 3 Faculty of Applied Mathematics, Silesian University of Technology, 44-100 Gliwice, Poland; 4 Baltic Institute of Advanced Technology, 01124 Vilnius, Lithuania

**Keywords:** self-administered cognitive testing, cognitive impairment detection, intelligent medical data analysis, clinical decision support, tactile sensing, biomedical signal processing, digital health

## Abstract

We present a model for digital neural impairment screening and self-assessment, which can evaluate cognitive and motor deficits for patients with symptoms of central nervous system (CNS) disorders, such as mild cognitive impairment (MCI), Parkinson’s disease (PD), Huntington’s disease (HD), or dementia. The data was collected with an Android mobile application that can track cognitive, hand tremor, energy expenditure, and speech features of subjects. We extracted 238 features as the model inputs using 16 tasks, 12 of them were based on a self-administered cognitive testing (SAGE) methodology and others used finger tapping and voice features acquired from the sensors of a smart mobile device (smartphone or tablet). Fifteen subjects were involved in the investigation: 7 patients with neurological disorders (1 with Parkinson’s disease, 3 with Huntington’s disease, 1 with early dementia, 1 with cerebral palsy, 1 post-stroke) and 8 healthy subjects. The finger tapping, SAGE, energy expenditure, and speech analysis features were used for neural impairment evaluations. The best results were achieved using a fusion of 13 classifiers for combined finger tapping and SAGE features (96.12% accuracy), and using bidirectional long short-term memory (BiLSTM) (94.29% accuracy) for speech analysis features.

## 1. Introduction

Degenerative disorders of the central nervous system (CNS) such as Huntington’s disease (HD), Parkinson’s Disease (PD), Alzheimer’s Disease (AD), and mild cognitive impairment (MCI) affect the human motor system and exhibit a set of similar deficits, such as cognitive impairment and motor dysfunctions [1]. Examples of such dysfunctions are a reduced speech rate [2], higher daily caloric intake [3], increased rigidity, reduced dexterity, and essential tremors [4]. Current studies focus on recognizing the early symptoms of the diseases such that future medical help may delay its progress [5]. The Self-Administered Gerocognitive Examination (SAGE) [6] is a commonly used tool of cognitive assessment of MCI and early dementia symptoms. SAGE is widely applied for assessing the symptoms and progress of MCI, AD, and PD [7]. Any auxiliary measure (e.g., a digital tool for health state self-assessment) created to improve the daily life of patients and medical doctors is beneficial.

Here we focus on the development of a computerized model and tool that assesses the cognitive and motor deficits of patients at the early stage of CNS disorders, thus helping in delaying the progression of disease symptoms and providing more years of healthy life. We propose a digitized data collection methodology via a smart electronic consumer device (tablet or smartphone) interface adapted for patients with CNS disorders, thus resulting in feature extraction for model inputs, and propose hybrid healthy vs. impaired person classification models for the tasks aimed at the evaluation of CNS degeneration symptoms.

Our scientific contribution is the digitization of the SAGE methodology for the detection of early signs in memory or thinking cognitive impairments to automatically evaluate the patient’s health state (without paper form and doctor supervision) and the extension of the SAGE methodology with extra tests for tremor and energy expenditure impairments.

The structural organization of the paper is as follows. Section 2 analyzes the related work. Section 3 covers the materials and methods used, i.e., the implementation of a computerized extended SAGE, methods for feature extraction and analysis, and classifier fusion. Section 4 describes four conducted classification experiments for cognitive impairment screening. Finally, Section 5 presents discussion and concluding remarks.

## 2. Related Work

Recently, many works have focused on providing digital cognitive and motoric function self-assessment tests on electronic consumer devices, such as smartphones [8], tablets [9], and dedicated graphical tablets [10]. Commonly implemented tests include Archimedes spiral drawing tasks [11,12,13,14,15,16,17], finger tapping [9], freehand drawing tasks [17], and tracing tasks [12,17]. The methods used to analyze the collected spatiotemporal finger tapping, finger drawing, or pen path data include statistical analysis [11,12,15], discrete cosine transform (DCT) features [10], entropy, and fractal dimension analysis [13].

Previous works on the neural impairment symptom diagnostics have employed a variety of methods, such as an artificial neural network (ANN) [18], radial basis function neural network (RBFNN) [19], dynamic neural network (DNN) [20], decision tree, ID3 [21], adaptive neuro-fuzzy [22], neuro-fuzzy system [23], fusion of classifiers (Bayesian, k-nearest neighbor (KNN), support vector machine (SVM)) [24], and neuro-fuzzy network [25]. Speech analysis has been used, including OpenSMILE features, Essentia descriptors, MPEG7 descriptors, KTU, jAudio, YAAFE, Tsanas audio features, and a random forest (RF) classifier to detect PD and to fuse features obtained from separate input modalities [26]. The cepstral separation difference (CSD) was applied to the evaluation of speech impairment in PD [27]. Feature extraction using the signal-to-noise ratio (SNR), harmonic-to-noise ratio (HNR), vocal fold excitation ratio (VFER), glottal to noise excitation (GNE), and empirical mode decomposition excitation ratio (EMD-ER) methods with random forest (RF) and SVM for classification were used in Tsanas et al. [28]. Other approaches were introduced in An et al. [29], namely syllable-level, low-level descriptor (LLD), formant, and phonotactic features with an SVM classifier and features from principal component analysis (PCA); while Caesarendra et al. [30] introduced linear discriminant analysis (LDA), SVM, adaptive boosting (AdaBoost), KNN, and adaptive resonance theory—Kohonen neural network (ART-KNN).

Energy expenditure impairments related to weight loss in HD are linked to the human genome and are likely the outcome of a hypermetabolic state [31]. HD patients often experience weight loss, mainly due to the negative energy balance, which may lead to weight loss even when their caloric intake was larger than that of healthy subjects [32].

## 3. Materials and Methods

### 3.1. Methodology

To develop the materials and methods, a data collection tool was proposed, based on a self-administered cognitive testing methodology (SAGE), which is used to identify the signs of MCI and early dementia in, e.g., Huntington’s disease, Parkinson’s disease, and Alzheimer’s disease patients. SAGE is applied in practice (mostly in the USA) by medical practitioner-neurologists by submitting questionnaires to patients in paper form and assessing their condition manually.

Additionally, methods for the feature extraction of tremor, cognitive, and energy expenditure impairments were defined: Euclidean and Frechet distances for spirography curve comparison, Jaro algorithm to compare the string input, neighbor matching for a graph similarity measure, the daily calories gained and burned calculation using the basal metabolic rate (BMR). For speech analysis, the following feature extraction methods were used: pitch, gammatone cepstral coefficients (GTCC), Mel-frequency cepstral coefficients (MFCC), wavelet scattering transform (WST), and the spectral analysis methods in the frequency domain.

### 3.2. Mobile Application

The intelligent mobile app developed for Android devices served as a digital health state evaluation tool for patients with neurological disorders (HD, PD, and MCI) based on cognitive, motoric, speech, and energy expenditure impairments. Two modes (training and testing) were available. In the training mode, a patient was instructed to try out different tasks as an exercise. In the testing mode, all tasks were integrated by giving a single task only once and providing each successive task in a random order. Such an approach ensured that the patient did not memorize all the questions when repeated test attempts were taken. When an actual test finished, the results were presented to the patient.

**Figure 1:**
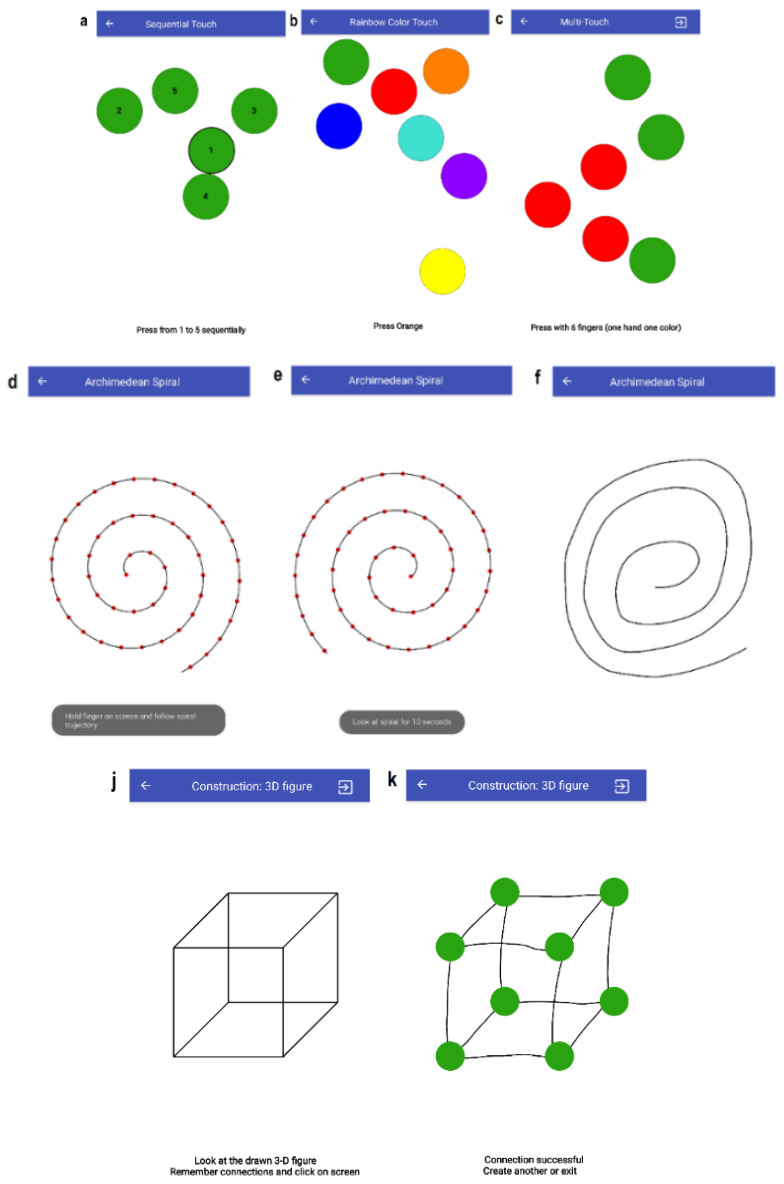
Screenshots of screening tasks: touches ((**a**)—sequential; (**b**)—rainbow color; (**c**)—multi-touch); Archimedean spiral ((**d**)—following contour clockwise; (**e**)—showing spiral counterclockwise; (**f**)—drawing contour counterclockwise); construction of 3D figure ((**j**)—showing; (**k**)—constructing cube).

Figure 1 illustrates the core functionality (tremor, cognitive, speech, energy expenditure tasks) for the data collection platform implemented as a mobile application of the smartphone or tablet, which is available free online as a self-assessment tool (available on Google Play at https://play.google.com/store/apps/details?id=com.alauraitis.test_suite&hl=en).

### 3.3. Tasks

The developed smart mobile app supports 16 different tasks (12 from the SAGE methodology and an extra 4 were added based on the related research) for the assessment of the patient’s health state and early screening of neural and motor impairments (see Table 1). All tasks (in particular, the T4—Archimedean spiral; T9—construction (3D figure); T10—construction (clock); T12—executive: modified trials; and T13—executive: problem-solving) that required direct interaction of finger movements of the subject were executed without using a smart pen. Such an approach ensured that there were no repercussions on the test results.

#### 3.3.1. T1–T3: Finger-Tapping Tasks

For the finger-tapping tasks (T1, T2, T3), the subject is instructed to touch the circular shape objects using a single finger (T1, T2) and multiple fingers (T3). The goal is to touch the displayed objects as quickly and accurately as possible.

In task T1, the circular objects (2, 3, and 5 at a time) are randomly placed on the mobile device screen. The subject must try to touch an active circle marked by a black contour.

In task T2, there are seven objects of different (rainbow) colors. The screen is redrawn 5 times, i.e., the subject needs to touch the object five times. Each time the colored circle that has to be touched is randomly selected and displayed. T2 attempts to challenge the HD subject more by providing a greater level of uncertainty on which object they must touch.

In T3, touching with multiple fingers on objects is required.

#### 3.3.2. T4: Archimedean Spiral

The Archimedean spiral contour was experimentally examined to be an indicator of detecting early signs in PD [33]. In this context, the PD and HD tremors are related such that T4 is adaptable as a significant measurement method of a tremor impairment state for the HD patients. In T4, the spiral is shown for 10 sec to the user in the device screen clockwise. The subject is instructed to follow the spiral contour with a finger. After that, the screen is cleared so that the subject can try to replicate the shape of a spiral contour with his finger.

We use the Frechet distance algorithm [34] to evaluate the difference between the predefined spiral and the curve drawn by the subject. Another method considers the percentage match by determining whether a subject’s touched point is within the radius (calculated using the Euclidean distance) of the closest point from the point set of the predefined spiral.

#### 3.3.3. T5–T7: SAGE Cognitive Tests

The implemented SAGE tests include a non-scored item, i.e., a basic questionnaire asking the subject about their demographic status, family history, problems with memory and thinking, depression, motor and stroke symptoms, functional abilities, and personality changes (T5).

In task T6, the subject is asked to enter the current date from their memory: year (Y), month (M), and day (D). For each valid answer, a subject receives one point and the points are accumulated.

In task T7, the subject is instructed to name two pictures for visual inspection. All pictures were collected from four SAGE forms (eight in total) such that the same images are used, as proposed by methodology creators. Moreover, the procedure was improved by adding enhanced full-color images and extending the image set with two extra pictures for a bigger surprise factor. Once the image set is associated with a randomly selected SAGE group, a single picture is randomly (first or second) selected for the subject to name. Next, the subject is provided with an input field for entering the text associated with the shown image. Navigation is also available, i.e., the subject can go to the previous or the next picture if an already-provided answer needs to be corrected. In the case when the subject knows the name of the object in a picture name but makes a spelling error (e.g., due to a tremor), another strategy is adapted. For inexact string matching, we apply the Jaro-Winkler (JA) algorithm [35] that outputs 1 for a perfect string match using a symbol-by-symbol comparison.

#### 3.3.4. T8: Similarities Calculation

Task T8 covers three questions (Q3, Q4, Q5) from SAGE.

In the first question (Q3), the subject is given a text query, which requires finding the similarity between two listed items (e.g., watch and ruler, or corkscrew and hammer). Two forms for answering are considered: (1) abstract; and (2) concrete. The maximum score for Q3 is given when the subject answers in an abstract manner, i.e., they manage to find an upper-level category to which both items can be classified.

In Q4 and Q5, the subject’s mathematical knowledge is tested. Question Q4 requires performing a mathematical subtraction operation of two floating numbers. The context of the Q4 question is about going to the grocery store and buying items for a specified money value M. The subject needs to calculate the change received back from the paid bill B (B > M, chosen randomly in an interval (0, 100]).

Question Q5 requires performing a mathematical division operation of two floating numbers. The context of Q5 is about having a sum of money S and coins of some denomination D (e.g., dime, nickel, quarter, etc.) and calculating how many coins will be needed to collect S. For Q4 and Q5, the subject cannot use a calculator (only their brain or a paper sheet).

As for methods used for the subject evaluation of T8, in Q3 we applied the exact and inexact string matching (including the Jaro-Winkler method). Correct answers for a single term have associated point values (2 for an abstract answer, 1 for a concrete answer). For the evaluation of Q4 and Q5, one point is given when a subject provides a correct answer.

#### 3.3.5. T9: 3D Construction

Task T9 covers the seventh question from SAGE. T9 asks the subject to reconstruct a given 3D figure (cube, ribbed rectangle). As defined in SAGE, there can be four different 3D figures: cube, rectangle, and variations (e.g., a cube missing a surface). After reading the instructions, the subject clicks on the mobile device screen and is redirected to the task execution mode. Here, eight graph nodes are displayed, but no edges between the nodes are visible because the task for the subject is to form proper connections. An edge can be formed in a free manner, i.e., a subject must draw a path with their finger on the screen between two nodes. We assumed that constructing the 3D figure in such way can trigger many errors (especially for subjects with hand tremor symptoms) such that these error metrics are computed: the number of wrongly connected nodes, the number of errors when too few nodes are selected when forming a connection, the number of errors when too many nodes are selected when forming a connection, and the number of errors when an already existing connection is selected.

Another aspect of evaluating the subject in task T9 is the calculation of the parallel line angles between the edge pairs in the constructed 3D figure. This is important because of the free path (trajectory) drawing that is performed. The parallel line angle calculation is done by taking the arctangent of two slopes (m1, m2) of the analyzed line pairs in the 3D cube. According to SAGE, the tolerance of parallel lines is 10. We adapted an extra tolerance match percentage factor (default is 50%) for an estimation of how many parallel line pairs (9 in total) were connected by an angle of less than 10°.

#### 3.3.6. T10: Construction (Clock)

**Figure 2:**
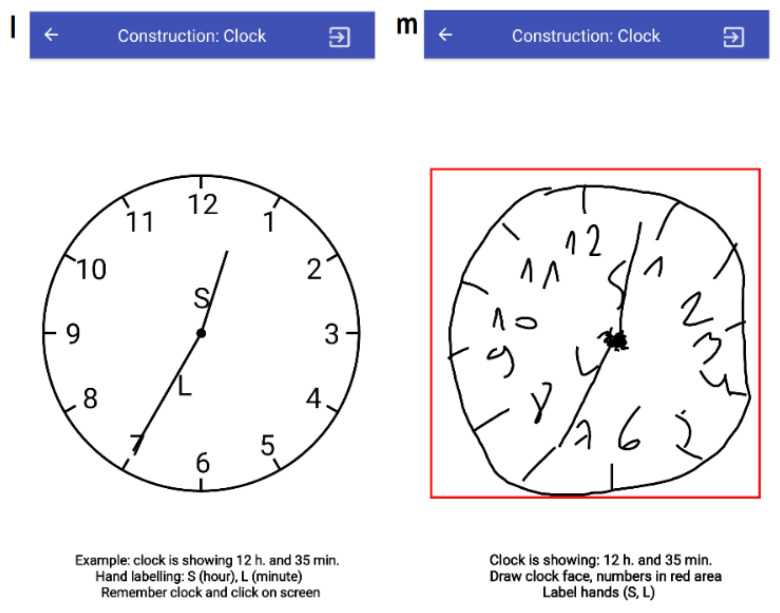
Screenshots of the task T10: clock construction (l—showing, m—constructing clock).

Task T10 covers the eighth question of the SAGE survey. In T10 (Figure 2), the subject is instructed to perform the clock drawing test (CDT) using their finger. First, in the preparation mode, an example analog clock showing the predefined hours (H, a random value in [1, 12]) and minutes (M, a random value in [0, 60], 5-min step) is displayed. The clock components are the clock face and all 12 numbers, hand positions, and hand labeling (S—hours, L—minutes). The subject is given instructions at the bottom of the screen to remember the clock. When a mobile device screen is clicked once more, the subject is redirected to the CDT execution mode. There, an active zone (contour) is provided for drawing the clock. If the subject violated the bounds of the active contour, an error is triggered. The subject’s evaluation of the CDT task is semi-automatic, i.e., a supervisor is required to assess the clock drawn.

#### 3.3.7. T11: Verbal Fluency

**Figure 3:**
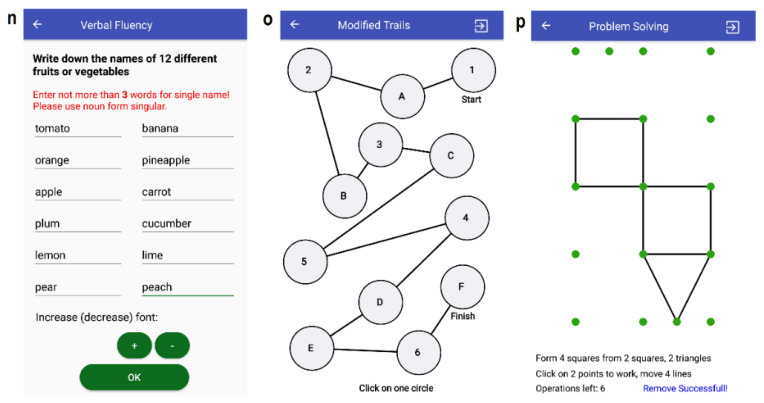
Screenshots of the mobile app tasks: T11: verbal fluency (n—entering 12 items); T12: modified trials (o—completing schema); T13: problem solving (p—after line remove operation).

Task T11 implements the ninth question from the SAGE survey. The subject is instructed to write down 12 different items (elements) in the given category (Figure 3). There are four different categories (associated with a SAGE group): animals, fruits or vegetables, objects that can be found in kitchen (not including food), and countries of the world. In the mobile app, twelve text fields are provided for subject input (not more than three words should be entered for a single name). The text field can be left blank in case the subject does not remember any items of the category.

The maximum SAGE score for T11 is 2 points (when a subject enters 12 different items), 1 point (when 10 or 11 correct items are entered), and 0 points (otherwise). In the case of inexact string matching (when the input term is not found in the dictionary), we used the Jaro-Winkler method to compare the symbol-by-symbol equivalent of the closest-found match from the dictionary. Finally, the average of all Jaro-Winkler calculations to 12 items is calculated (for a perfect match, the total average is 1).

#### 3.3.8. T12: Executive (Modified Trials)

Task T12 corresponds to the 10th question from SAGE. The subject is asked to follow a pattern in the schema (Figure 3), i.e., to draw a line from one circle to another, while alternating the numbers and the letters (e.g., 1-A-2-B-3-C is a valid sequence). To form a connection between two nodes, the subject uses their finger in a free-drawing form (similarly to task T9) to create path trajectories. When a finger is released, the path is processed and successfully formed only if exactly two nodes have been touched (as measured by the Euclidean distance between two points). The following measures are calculated: the number of wrongly connected nodes, the number of errors when clicking on the same node, and the number of clicks outside a node. Additionally, the graph similarity metric between the predefined and subject schemas is calculated using the neighbor-matching algorithm. Furthermore, the Frechet Distance is calculated with respect to the linear interpolation to each properly formed path, e.g., between a smooth line connecting the node “1” to the node “A” and the subject’s drawn path between the nodes “1” and “A”.

#### 3.3.9. T13: Executive (Problem Solving)

Task T13 corresponds to the 11th question from the SAGE survey. It implements a simulation of a simple game, which requires performing movements to form a new shape from a given shape by moving some of its edges represented by matches (Figure 3). The SAGE methodology has four geometrical shape transformation tasks. All these problem-solving tasks were integrated into the developed mobile application. When a subject starts to move edges, only two types of operation are allowed: insert and remove. To insert a line, two nodes (with no connection between them) need to be clicked sequentially on the screen. To remove a line, two nodes (with a connection between them) should be touched sequentially. An operation is successfully executed when the touching of two nodes occurs inside of each node (the deviation is calculated using the Euclidean distance). Similarly, as in T9 and T12, we assumed that the subjects with neurological disorders can make many different errors, such as touching the same node twice, touching the node outside of the zone of operation, and violating the rules for allowed operations, e.g., trying to execute the insert operation, when only the delete operation is allowed. For this reason, we adapted a numerical error-tracking mechanism. Task T13 finishes when the number of allowed operations left is equal to 0 or a special button is clicked in the action bar. The evaluation of task T13 is similar to task T9, i.e., the neighbor-matching algorithm for calculating the graph similarity measure is adopted.

#### 3.3.10. T14: Voice Recorder

Task T14 instructs a subject to read a short text from the pre-defined poems into the microphone of a mobile device. The process is repeated two times with different poems selected randomly for a more reliable execution of the test. The Neural Impairment Test Suite app was developed in Android SDK, therefore audio files were stored in mpeg4 format based on the compatibility requirements with the latest version of Android. The MPEG-4 audio codec was set at a standard sampling rate of 48 kHz (mono) and 16 bits. This provided an exceptional audio quality considering the nature of the recording (speech can be analyzed even in lowly LPCM 16 bit, 16 kHz formats). The compression (128 kbps) had no measurable effect on the quality of the audio records used for the analysis. All of the recordings were double-checked by an expert to verify the quality of the T14 task. During the controlled experiments, isolation of the surrounding environment noise was ensured while the 60 cm distance from the speaker’s mouth to the mobile device was maintained.

We used the following methods for the extraction of speech features: Pitch was used for estimating the fundamental frequency of the input audio signal with a 44.1 kHz sampling rate. The pitch contours were estimated using the normalized correlation function [36], pitch estimation filter [37], cepstrum pitch determination [38], log-harmonic summation [39], summation of residual harmonics [40], and Mel-frequency cepstral coefficients (MFCC) [41]. Gammatone cepstral coefficients (GTCC) is a perceptual modification of MFCC that uses the gammatone (GT) filters [42]. The GT filter bank was applied to the signal’s coefficients of a fast Fourier transform (FFT), focusing on the perceptually meaningful sound frequencies. Finally, the discrete cosine transform (DCT) was applied. We also used a wavelet scattering transform (WST), which is based on the stages of wavelet decompositions and modulus operators [43].

We adopted the following feature extraction methods from voice signals and voice spectrograms: spectral slope [44], spectral skewness, spectral centroid, spectral spread, spectral decrease, spectral kurtosis [45], spectral flux and spectral rolloff [46], spectral flatness [47], and spectral entropy [48]. The spectral slope measures the slope of the shape of a spectrum using linear regression. The spectral skewness measures the symmetry of the distribution of the spectrum values around their average value. The spectral spread evaluates the distribution of the power spectrum around the spectral centroid. The spectral centroid evaluates the center of gravity of spectral energy. The spectral decrease measures the steepness of the spectral envelope decrease vs. the change of frequency. The spectral kurtosis evaluates the similarity of the distribution of its spectral magnitude values to a normal distribution. The spectral flux assesses the transformation of the spectral shape by comparing the consecutive short-time Fourier transform (STFT) frames. The spectral rolloff assesses the bandwidth of the audio samples in terms of the accumulated magnitudes of the STFT to reach a certain threshold. The spectral flatness is the ratio of geometric mean and arithmetic mean of the magnitude spectrum. The spectral entropy captures the distribution of spectral power.

#### 3.3.11. T15: Total Daily Energy Expenditure (TDEE)

In task T15, first, information about the subject is collected: gender (G—man or woman), age (A, in years), height (H, in m), weight (W, in kg), and physical activity level (PAL—sedentary, lightly active, moderately active, very active, or extremely active), while the body fat percentage is left optional. Based on these five parameters, the basal metabolic rate (BMR) and total daily energy expenditure (TDEE) are calculated using the Mifflin St Jeor formula [49]:

(1) $$BMR=\{\begin{array}{c} \begin{array}{cc} \left(625\cdot H\right)+\left(10\cdot W\right)-\left(5\cdot A\right)+5, & \mathrm{men} \end{array}\\ \begin{array}{cc} \left(625\cdot H\right)+\left(10\cdot W\right)-\left(5\cdot A\right)-161, & \mathrm{women} \end{array} \end{array},$$

(2) TDEE=BMR·PAL,

where *BMR*—Basal Metabolic Rate, *PAL*—physical activity level (sedentary, light (1–3 days per week), moderate (3–5 days per week), heavy (6–7 days per week), athlete (2 times per day)), *H*—height (m), *W*—weight (kg), *A*—age (years).

The next part of task T15 covers tracking the subject’s daily gained and burned calories using two modes: second (“analyze daily food”) and third (“enter performed physical activities”). In the second mode, a subject is instructed to remember what food they ate during a day, e.g., a single food item is entered using this approach: product name (picked from a predefined list with auto-completion functionality), quantity (in grams), and mealtime (breakfast, dinner, and supper). The product list is used to calculate calories gained from a single entered product item. The subject can enter as many food items as they can remember. The total daily gained calories for a subject are calculated using:

(3) $$P_{\mathrm{gained}}=\sum_{i=1}^{n}\frac{m_{\mathrm{patient}}\cdot Cal_{100}}{m_{\mathrm{norm}}},$$

where $P_{\mathrm{gained}}$—subject’s daily gained calories (from food), n—number of products the subject input, $m_{\mathrm{subject}}$—input product quantity (in grams), $Cal_{100}$—input product calorie norm (for 100 g), and $m_{\mathrm{norm}}$—value of 100 (grams).

In the third mode, the subject is instructed to remember what physical activities (e.g., doing exercises) they performed during a day. A single physical activity is entered as follows: physical activity name (picked from a predefined list with auto-completion functionality), duration (minutes), and time of day (morning, day, evening). The activity list, associated with relevant metabolic equivalent task (MET) values, is created using National Cancer Institute data [50]. The total daily burned calories are calculated using:

(4) $$P_{\mathrm{burned}}=\sum_{i=1}^{m}\frac{MET_{i}\cdot W\cdot D}{60},$$

where m—number of subject activities, $MET_{i}$—MET coefficient of the item i, D—activity duration in minutes, $P_{\mathrm{burned}}$—the subject’s daily burned calories, and W—the subject’s weight (in kg).

Finally, the daily calorie balance ($P_{\mathrm{balance}}$) for a subject is calculated using:

(5) $$P_{\mathrm{balance}}=P_{\mathrm{gained}}-P_{\mathrm{burned}}.$$

#### 3.3.12. T0: Memory

In task T0, the subject is instructed to memorize a phrase that was asked to remember before the test process. SAGE proposes using two phrases: “Have you finished?” and “Are you done?”. A maximum of two points are given when the exact wording (no extra wording) is provided, 1 point if a word (“finished” or “done”) is found in the subject’s input text, and 0 points otherwise.

### 3.4. Hybrid Classification Model for Decision Support

To provide decision support, we investigated the following classification methods: SVM, artificial neural networks (ANNs) with a multilayer perceptron (MLP), K-nearest neighbors (KNNs), sequential minimal optimization (SMO) [51], linear discriminant analysis (LDA) [52], Fisher’s linear discriminant analysis (FLDA) [53], deep learning networks (DNNs), random forests [54], Bayes nets [55], naive Bayes [56], decision tree (J48) [57], stochastic gradient descent for applied to linear models (SGD) [58], logistic model trees (LMTs) [59], decision stump [60], and voted perceptron [61]. Moreover, the boosting algorithms [62] were adapted for classifier ensemble learning.

The motives for choosing a classifier ensemble (multiple models) for making the final decision were the intuition and hypothesis that such a hybrid classifier increases the model accuracy and decreases occurrences of the model’s errors. Moreover, aggregating several different classifiers was expected to bring the resultant classifier to the one that better fits the problem [63].

For feature reduction, we analyzed four methods: principal component analysis (PCA) with a variance covariance (VC) configuration parameter in the original data [64], correlation attribute evaluation [65] for the evaluation of attribute relationships to the target class, wrapper subset evaluation (WSE) [66], and classifier attribute evaluation (CAE). The considered attribute search methods were: best first (forward, backward, greedy) and ranker [67]. Finally, for the combination of classifiers and the hybridization, we used the voting method [68] with the average of probabilities (AP) combination rule.

**Figure 4:**
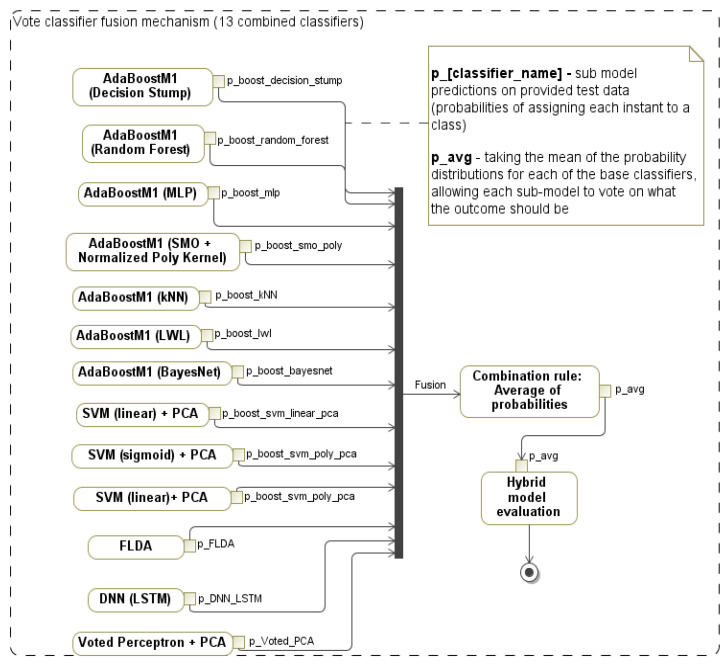
Proposed hybrid classification model that combines 13 classifiers for the detection of tremor, cognitive, and energy expenditure impairments.

For the hybrid classification, we combined the outputs at the classification level, which resulted in a hybrid model. The best results from the single classifiers were taken for the fusion mechanism. The same model could be used many times (e.g., Figure 4 shows that the combination of SVM (linear) and PCA was applied twice) if needed.

The stand-alone classifier models were combined using the majority vote and “average of probabilities” combination rule (*p*_avg). In such a scenario, each classifier generated predictions (*p*_classifier_name) using the provided testing data (probabilities of assigning each data instance to a class), then taking the mean of the probability distributions for each of the base classifiers and allowing each sub-model to vote on what the outcome should be. The majority vote method is defined as an ensemble decision for the class.

### 3.5. Hardware

The mobile app was tested on several mobile devices with different screen size and resolution characteristics: OnePlus 5 (5.5″ screen with a resolution of 1920 × 1080 px), SAMSUNG S7 (5.1″, 2560 × 1440 px), and Lenovo YOGA YT3-X50L (10.1″, 1280 × 800 px). The recommended device for carrying out tests was with a bigger screen size (in this case, the YOGA YT3-X50L tablet) because the subjects who had tremor impairments had more difficulties when touching the screen positions accurately. Moreover, the older test subjects had weaker vision and less experience with using mobile devices.

### 3.6. Data Collection

The assistant should help the subject to perform the test for the first time by explaining the working principles of each task. In later stages, if the state of health allows, the subject can work individually using their own mobile device or can be helped by family members or medical personnel. However, a supervised procedure is recommended to ensure the correct execution of the test. The duration of the test with a single subject is about 20 min. Multiple test attempts are required to evaluate the possible progression of the subject’s neurological disorders over time. A subsequent attempt should consider the time scale, e.g., within approx. 2–3 weeks (or up to 1 month) after the previous execution of the test.

### 3.7. Subjects

Fifteen test subjects participated in the investigation. Seven patients with neurological disorders (three with Huntington’s disease, one with Parkinson’s disease (senior male, 74 years old, contribution to the dataset was 10 records), one with cerebral palsy (teenage male, 20 years old, contributed 7 records), one post-stroke (adult male, 60 years old, contributed 8 records), one early dementia (adult female, 40 years old, contributed 14 records)); the other eight were healthy subjects. All three HD patients were males, one of them was a juvenile of 18 years (contributed 4 records), while the other two were adults aged 42 and 44 years, respectively (20 records from each). Regarding the healthy subjects, there were four males (two of them were 33 years (collected 22 records from each), one was 42 years (10 records), and one was 65 years (7 records)) and four females (two of them 60 years (29 records obtained from each), one was 20 years (11 records), and one was 26 years (7 records)). An informed consent form was signed by all subjects.

All participants performed the same sets of tasks (16 in total) considering that the health states of the patients with CNS disorders were in their early stage, i.e., stage I or II based on the Shoulson-Fahn scale [69]. The main symptoms of such patients were balance disorders, hand tremors, the development of an early negative energy balance, body and muscle stagnancy, decision-making problems, focus attention issues, and memory loss.

### 3.8. Dataset

The collected dataset had 150 records (89 records taken from healthy control subjects and 61 samples taken from patients with CNS disorders). Each record had 238 features acquired during 5 rounds (i.e., the visitations of a subject), where each round contributed ≈30 entries to the dataset). All visitations were carried out using face-to-face communication with patients by providing direct supervision for test execution. The data was collected in five rounds in 2019: first round (20 February–21 March), second round (10 April–21 April), third round (7 May–16 May), fourth round (3 July–1 August), and fifth round (2 September–10 September). In each round (five times in total for each subject), the full testing procedure (all tasks were considered) was conducted. No significant learning effect was observed in the patients with CNS disorders nor the healthy subject control group as all test subjects tended to feel strained during the testing procedure.

## 4. Experiments and Results

### 4.1. Outline

To validate our methodology and developed digital tool, we performed four experiments:

1. Experiment 1 (E1): the feature set is distributed using individual tasks only (14 different classifiers).
2. Experiment 2 (E2): all 238 features were combined (integrated) and fed into a classifier, were combinations of classifiers were used to propose a hybrid model.
3. Experiment 3 (E3): audio files (from task T14) were used for further processing to extract features using a combination of methods (pitch, MFCC, GTCC, and spectral skewness) and to classify samples with deep learning networks (in particular, bidirectional long short-term memory (BiLSTM)).
4. Experiment 4 (E4): audio files (from T14) were used for further processing to extract features using the WST method and to classify samples with an SVM.

The results of experiments E1 and E2 were analyzed with WEKA (University of Waikato, Hamilton, New Zealand), whereas experiments E3 and E4 were analyzed with MATLAB Audio Toolbox R2019a (Mathworks Inc., Natick, MA, USA).

### 4.2. Cross-Validation

In E1 and E3, the 10-fold cross-validation procedure was used.

In E2, for validation of models using unseen data, the dataset of all collected records was split into 129 records for training (N-cross validation procedure) by omitting records of three randomly chosen patients (one with juvenile Huntington’s disease, one with Parkinson’s disease, one with MCI) and three healthy test subjects. These omitted records were supplied as two individual test sets (one for healthy (H), one for sick (S)) for predictions using new (unseen) data samples (healthy vs. sick classification). In such a setup, no mixed data from the same subjects in the training and testing sets existed.

In E4, the dataset was split into: (a) the training set (70%) consisting of 190 records (107 records from healthy subjects and 83 records from patients with CNS disorders, obtained from 6 healthy subjects and 5 patients with CNS disorders) and (b) the testing set (30%) consisting of 81 records (46 records from healthy subjects and 35 records from patients with CNS disorders, obtained from 2 healthy subjects and 2 patients with CNS disorders).

### 4.3. E1: Sick vs. Healthy Classification Models for the Individual Tasks

The experiment E1 was designed for triggering a screen alert based only on a single task executed by a test subject (impaired or healthy). Additionally, E1 was innovative for uniquely composed feature combinations for the determination of the health state evaluation.

Table 2 provides the best results for each task.

A higher accuracy indicates that the model could distinguish between two target classes better. For example, the logistic model tree (LMT) classifier, combined with PCA, resulted in a 91.50% accuracy for the T9 task. Other classifiers achieved slightly lower results (the lowest ones were spelling and T11 with 74.52%), which means that either extra indicators (evaluation criteria) were needed or more data should be used for training the model. If a classifier is in brackets with another classifier, the wrapper subset evaluation (WSE) method was applied for training the selected attributes. Model building speed on a CPU is provided in seconds.

### 4.4. E2: Impaired vs. Healthy Classification Models for the Integrated Feature Set

To avoid overfitting, when the number of features is larger than the number of samples, first, we applied binary grey wolf optimization particle swarm optimization (BGWOPSO) [70], which is a recently proposed hybrid feature selection method that was proven to be superior over a wide range of feature selection algorithms, to find the best feature subset. Feature selection resulted in the optimal set of 19 features, which included 3 features from task T1, 8 features from task T4, 6 features from task T14, and 2 features from task T15.

The dataset of all collected records for E2 was split for training (using 10-fold cross-validation) by the omitting the records of three randomly chosen patients (one with juvenile Huntington’s disease, one with Parkinson’s disease, one with MCI) and three healthy test subjects. These omitted records were supplied as two individual test sets (one for healthy (H), one for sick (S)) for predictions using unseen data samples (healthy vs. impaired classification). The boosting algorithm AdaBoostM1 [62] was used for tuning the classifier accuracy.

The evaluation metrics used for designed the models were: true positive rate (TPR); false positive rate (FPR); precision; recall (same as TPR); F1 score (F-measure); AUC (area under receiver operating characteristics (ROC), i.e., a plot of true positives vs. false positives for all potential cutoffs for a representation); Matthews correlation coefficient (MCC); and PRC (precision–recall plot, which is better adapted for imbalanced datasets).

Table 3 shows a comparison of the best results using TPR (sensitivity), TNR (specificity), precision, F1, and MCC metrics with 12 classifiers (as well as their combinations with boosting algorithms or attribute selection methods) for the E2 experiment. The best accuracy of 96.12% (using 10-fold cross-validation) was observed for the proposed hybrid model. Such a hybrid model generated 124 correctly classified test records (77 of which were from target class = 0 and 47 of target class = 1) and 5 incorrectly classified instances (2 of which were from target class = 0 and 3 of target class = 1). The hybrid model was also evaluated using the kappa metric (K = 0.918), root mean squared error (RMSE = 0.198), mean absolute error (MAE = 0.074), root relative squared error (RRSE = 40.741), and relative absolute error (RAE = 15.641).

To test the model using unseen data, the investigation of classifying new data samples was executed (see Table 4). For evaluation, we used prediction confidence (PrC, 0 ≤ PrC ≤ 1), i.e., the probability for a classifier to output an instance value, and an error count (EC), which indicates how many false predictions (alarms) occurred on sick and healthy test sets. Not a single classifier performed without an error (0 ≤ EC ≤ 2) on the healthy test set, but on the sick test set, the classifiers AdaBoostM1 (random forest), AdaBoostM1 (MLP), AdaBoostM1 (SMO), AdaBoostM1 (kNN), SVM (linear) + PCA, FLDA, DNN (LSTM), and voted perceptron + PCA worked without error (EC = 0).

### 4.5. E3: Speech Impairment Detection Using BiLSTM

The purpose of E3 was to classify voice recordings (64 kbps audio files in mp3 format), taken from the T14 task, into the impaired and healthy classes, thus building a model to predict suspected speech impairments for a subject. To eliminate silence segments that did not contain useful information on the health condition of the speaking person, the isolation of speech segments using the thresholding method was applied, which is described in more detail in Lauraitis et al. [71].

In E3, the training dataset and test set had 234 and 35 voice recordings, respectively (each test subject had their voice recorded multiple times). These feature sequences were normalized (using a z-score transformation) and used for training a bidirectional long short-term memory (BiLSTM) neural network [72]. BiLSTM was selected because the neural network could learn long-term associations between time steps of the sequential data, both in the forward and backward directions.

The BiLSTM architecture consisted of two fully connected network layers with 100 neurons each, succeeded by a softmax layer and a classification layer. For the training of the BiLSTM network, we used an RMSProp optimizer with a maximum of 10 epochs, a mini batch size of 128, shuffling on every epoch, a learning rate drop factor of 0.1, and the “piecewise” learning rate schedule. The parallel pool was used (with four workers) for training BSTM to speed up the training process on a single GPU.

**Figure 5:**
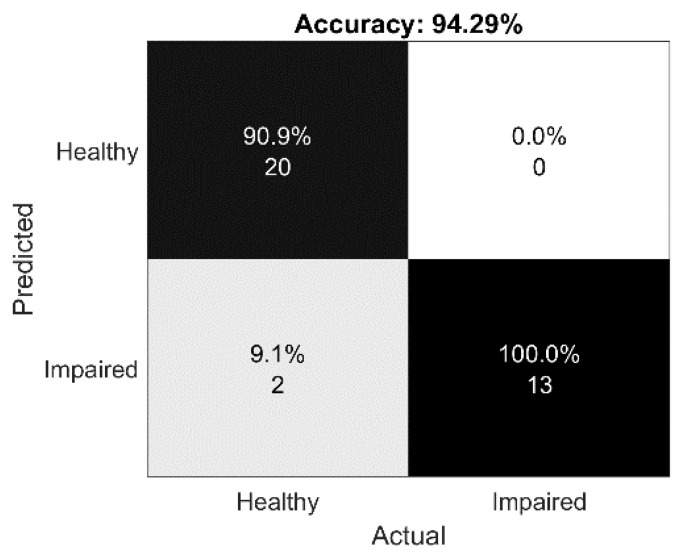
Confusion matrix of the E3 classification on 35 samples on a test set (20 correctly classified records (target class = 0), 13 correctly classified test records (target class = 1), 0 incorrectly classified instances (target class = 0), and 2 incorrectly classified instances (target class = 1)) using a bidirectional long short-term memory (BiLSTM) network and majority vote method.

Figure 5 illustrates the results of the impaired vs. healthy classification on the collected dataset after feature fusion and applying the majority vote rule for tuning the classifier performance. All instances from the training set were correctly classified, whereas in the testing set, only two healthy instances were incorrectly classified, thus giving an accuracy of 94.29%.

### 4.6. E4: Sick vs. Healthy Classification Using the Wavelet Scattering Transform Method (WST)

The purpose of E4 was the same as in E3, i.e., to solve the binary classification problem (impaired vs. healthy) based on speech data acquired from the T14 task. The WST method applied dilated Gabor (analytic Morlet) wavelets [73] with different scaling levels. The remaining parts of the scattering process were performed by convolving the input signal with the dilated wavelet. Other coefficients were computed based on this procedure: the fb1 (eight wavelets per octave) and fb2 (one wavelet per octave) wavelet filter banks were used, while the scale of time invariance was set to 0.5 s. Then, the scattering features were generated by applying a log transformation and setting the number of scattering windows to 8. The scattering features were used to train the SVM model with a third-order polynomial kernel, and a majority vote (as in E3) was applied, which allowed for achieving a perfect 100% accuracy using the test data.

### 4.7. Limitations

For subjects with cognitive and motor impairment (especially, for elderly individuals who are willing to carry out the proposed tasks day by day), it may be difficult and uncomfortable to perform some of the implemented tasks, such as the clock drawing, the TDEE, write down every time they eat something, or when they perform some physical activity. Therefore, they must receive appropriate supervision and guidance from the caregivers or family members to perform the self-assessment procedure effectively. For this reason, we recommend performing the full testing procedure (all 16 tasks) every 2–3 weeks (the insight is based on data collection pilot study findings from 15 test subjects considering 5 collection rounds). The performance of some of the tasks may be difficult on the devices with a small screen size; therefore, the use of the tablets with a larger screen size (at least 10″) is recommended. Using a smartphone with a small screen size may lead to reduced evaluation accuracy. Additionally, further usability considerations for the developed NITS mobile application are as follows: the mobile device has to have Android SDK v.6.0 or better (software part) with built-in functionality; support for an accelerometer, gyroscope, and finger pressure sensors; and a microphone for audio recording (device hardware part).

The voice features, such as MFCCs, are not primarily designed for the evaluation of dysarthric speech since they can be easily influenced by an individual speakers’ characteristics (gender, age, accent, etc.) or even characteristics’ of the microphone or sampling frequency. The results of voice cepstral analysis may be sensitive to specific speakers’ characteristics influenced by gender and age [74]. Furthermore, the limitation of the E3 and E4 experiments in the speech impairment detection was that the same subject could have been included in the training and testing datasets as several tasks for voice reproduction were performed.

The results may have been influenced by a small sample size because Huntington’s disease is very rare and there are only a handful of people with Huntington’s disease in Lithuania. A small sample size with a large number of features, such as in experiment E2 (238 features), could lead to overfitting. The small number of individuals participating in this study resulted in a biased dataset; however, combining different types of supervised learning classifiers in the hybrid model proposed in this paper allowed for increasing the overall classification accuracy by ≈2%. However, our experiment E1 considered only a small number of features for the collected dataset to classify healthy vs. impaired test subjects based on an individual task. Moreover, a technique of “early stopping” [75] was applied for individual classifier training to ensure there was no overtraining of the proposed models. For experiment E2, we adopted a feature selection method to reduce the number of features in the optimal feature set to 20.

## 5. Discussion and Concluding Remarks

We have presented an innovative health state monitoring approach using a smart mobile application “Neural Impairment Test Suite” (NITS), available on Google Play, which was specifically created for subjects who are suffering from neurodegenerative disorders and is aimed for day-to-day monitoring of their health state. The implemented system is based on a priori knowledge collected from medics and summarized as the SAGE (self-administered cognitive testing) methodology. A computerized version of the SAGE test was created, including the automatic evaluation of predefined scores for individual tasks. The extension of the methodology (extended SAGE) was implemented by adding finger tapping and speech impairment analysis tasks, and a component for visual observation of the subject’s health state and comparison with the results of self-assessment.

The developed mobile app is an actual framework for collecting 238 features of data. The proposed NITS framework requires only one smart non-invasive interface, i.e., mobile device or tablet for the neural impairment screening of subjects, thus proposing a convenient innovative health-state monitoring approach. Four experiments were carried out to solve the impaired vs. healthy binary classification problem: E1 (each task), E2 (all features), and E3 and E4 (voice recordings). For all four experiments, feature selection methods were applied by adapting a nested approach for cross-validation iterations in the model training. The collected dataset was used to validate the proposed experiments. E1 showed the best results for task T9 with the LWL classifier (91.50%). In E2, integrating a set of all extracted features (as defined in the extended methodology) and boosting classifiers with an ensemble learning AdaBoostM1 boosting method with the KNN classifier gave an improved accuracy of 94.57%. In addition, by fusing 13 classifiers (AdaBoostM1 (decision stump), AdaBoostM1 (random forest), AdaBoostM1 (MLP), AdaBoostM1 (SMO), AdaBoostM1 (kNN), AdaBoostM1 (LWL), AdaBoostM1 (Bayes net), SVM (sigmoid) + PCA, 2 × SVM (linear) + PCA, FLDA, DNN (LSTM), and voted perceptron + PCA) with the vote method (using the average of probabilities combination rule) resulted in a hybrid model with an improved accuracy of 96.12%. For the speech features, RNN with LSTM achieved an accuracy of 94.29% for a test set. The wavelet scattering transform (WST) achieved an accuracy of 100% for a test set of speech features. The insight observed was that in the feature integration (E2) experiment compared to the feature distribution experiment (E1), the classification accuracy improved, supporting the assumption that the fusion of features from several executive tasks improved the diagnosis accuracy.

The speech analysis results can be related to the computerized SAGE test by triggering a separate alert in the proposed NITS application and showing the results of possible speech impairment after the execution of the test procedure. Such an approach enables multiple alarm-tracking systems for decision support on the health state evaluation of the patient with CNS disorders.

The proposed smart computer-aided, self-administered testing extended methodology can be compared with several recent works of other researchers working on the digitalization of neuropsychological tests on tablets. The computerization of a Trail Making Test via mobile application proposed in Dahmen et al. [76] has its limitations as only one task is computerized (it corresponds to the T12 task in the proposed NITS framework) and only cognitive impairments are considered. An extension of the computerized Mental State Examination methodology proposed in Impedovo et al. [77] uses a specialized device-tablet (Wacom MobileStudio Pro 13) for data collection and achieved 93.3% accuracy while using SVM, discriminant analysis, convolutional neural network, and naïve Bayes classifiers.

The developed classification models cover a very wide range of possible disorders, encompassing subjects suffering from Huntington’s disease, Parkinson’s disease, or MCI: one can have a tremor in the hands or body, others may have memory loss, voice problems, or weight loss. This implies a high potential for the determination of the deteriorating status of each subject’s health state for early screening and disease progress monitoring. The models were integrated into a smart mobile application, which allows for daily monitoring of the disease’s progress. Another innovative aspect proposed in this paper is a new dataset, thus opening a gateway for using it in the machine learning repositories (such as in University of California Irvine (UCI) Machine Learning Repository) by other researchers.

## Figures and Tables

**Table 1:** List of all available tasks in the mobile app.

No.	Task Name	Impairment to be Addressed
T1	Sequential Touch	Tremor, Cognitive
T2	Rainbow Color Touch	Tremor, Cognitive
T3	Multi-Touch	Tremor, Cognitive
T4	Archimedean Spiral	Tremor, Cognitive
T5	Insights	Cognitive
T6	Orientation (current date)	Cognitive
T7	Picture Naming	Cognitive
T8	Similarities, Calculation	Cognitive
T9	Construction (3D figure)	Cognitive, Tremor
T10	Construction (clock)	Cognitive, Tremor
T11	Verbal Fluency	Cognitive
T12	Executive: Modified Trials	Cognitive, Tremor
T13	Executive: Problem Solving	Cognitive, Tremor
T14	Voice Recorder	Speech
T15	Total Daily Energy Expenditure (TDEE)	Energy Expenditure
T0	Memory	Cognitive

**Table 2:** Healthy vs. impaired classification results of individual tasks.

Task: Features	Attribute Selection	Accuracy (10-Fold Cross-Validation)	Speed (s)
T1: 9	PCA (VC = 0.85)	J48:84.90%, SVM (RBF): 84.90%	Instant
T2: 10	WSE (VC = 0.60)	J48 (NBM): 81.13%	0.07
T3: 28	WSE	RF (KNN): 77.35%	1.72
T4 (spiral following): 22	WSE	LR (LDA): 84.90%,	1.49
		ANN (RF): 82.07%	36.06
T4 (spiral drawing): 22	WSE	ANN (KNN): 87.73%	2.16
		RF (FLDA): 86.79%	1.24
T9: 30	PCA (VC = 0.75) CAE	LMT: 91.50%	0.04,
		ANN: 90.56%	0.07
		RF: 90.56%	0.02
T10: 24	CAE WSE	KNN: 90.56%	Instant
		ANN (KNN): 89.62%	2.36
T11: 2	CAE	RF: 74.52%	0.02
T12: 33	CAE WSE	RF: 83.09%	0.03
		ANN (KNN): 82.07%	5.84
T13: 25	WSE	J48 (FLDA): 83.96%	0.50
T15: 4	CAE	SVM (RBF): 78.3%	Instant
Spelling (T7, T8, T11): 3	WSE	LMT (RF): 74.52%	2.76
SAGE (T6, T7, T8, T9, T10, T11, T12, T13, T0): 10	PCA (VC = 0.50) CAE	LMT: 84.90%	0.01
		SMO: 84.90%	0.03
Duration (all tests): 16	WSE	FLDA (FLDA): 89.62%	0.66

PCA (VC)—Principal component analysis with variance covered (VC); WSE—Wrapper subset evaluation; CAE—Correlation attribute evaluation; RF—Random forest; KNN—K-nearest Neighbor; SVM—Support vector machine; RBF—Radial basis function; LR—Logistic regression; NBM—Naive Bayes multinomial; LMT—Logistic model trees.

**Table 3:** Healthy vs. sick classification results for all features (accuracy metrics and speed, 10-fold classification): best values are shown in boldface.

Classifier	Accuracy (%)	TPR (Sensi-Tivity)	TNR (Speci-Ficity)	Precision	F1	MCC	ROC	PRC	Speed (s)
AdaBoostM1 (decision stump)	93.02	0.930	0.919	0.930	0.930	0.850	0.986	0.987	0.11
AdaBoostM1 (random forest)	94.57	0.946	0.922	0.948	0.945	0.887	0.990	0.990	0.13
AdaBoostM1 (MLP)	92.48	0.922	0.914	0.922	0.922	0.837	0.971	0.971	19.46
AdaBoostM1 (SMO)	92.24	0.922	0.922	0.923	0.923	0.838	0.967	0.967	0.36
AdaBoostM1 (kNN)	94.57	0.946	0.929	0.946	0.945	0.886	0.933	0.918	0.03
AdaBoostM1 (LWL)	91.47	0.915	0.909	0.915	0.915	0.821	0.972	0.973	13.62
AdaBoostM1 (Bayes net)	93.79	0.938	0.917	0.939	0.937	0.869	0.958	0.962	0.31
SVM (sigmoid) + PCA	91.47	0.915	0.909	0.915	0.915	0.821	0.912	0.881	0.24
SVM (linear) + PCA	92.24	0.922	0.914	0.922	0.922	0.837	0.918	0.890	0.23
FLDA	92.24	0.922	0.900	0.923	0.922	0.836	0.976	0.978	2.94
DNN (LSTM)	94.57	0.946	0.944	0.946	0.946	0.886	0.987	0.987	2.80
Voted perceptron + PCA	93.02	0.930	0.919	0.930	0.930	0.853	0.937	0.92	0.30
Hybrid (proposed by authors)	96.12	0.961	0.953	0.961	0.961	0.918	0.983	0.984	0.59

**Table 4:** Evaluating predictions using unseen data. Two individual test sets were considered: 10 samples were taken from healthy test subjects and 10 samples were taken from sick test subjects. The average PrC_0 from 10 samples (healthy test set, target class = 0), the average PrC_1 from 10 samples (sick test set, target class = 1), the EC_0 (healthy test set, target class = 0), and EC_1 (sick test set, target class = 1) were found.

Classifier	PrC_0	PrC_1	EC_0	EC_1
AdaBoostM1 (decision stump)	1	0.934	1	1
AdaBoostM1 (random forest)	0.902	0.736	1	0
AdaBoostM1 (LMT)	1	1	1	0
AdaBoostM1 (ANN-MLP)	0.999	0.997	1	0
AdaBoostM1 (SMO)	1	0.996	1	0
AdaBoostM1 (kNN)	0.992	0.992	1	0
AdaBoostM1 (LWL)	1	0.972	1	1
AdaBoostM1 (Bayes net)	1	0.995	1	1
SVM (sigmoid) + PCA	1	1	1	2
SVM (linear) + PCA	1	1	1	0
FLDA	0.523	0.523	1	0
DNN (LSTM)	0.998	0.996	1	0
Voted perceptron + PCA	1	1	2	0
Hybrid (proposed)	0.991	0.931	1	0
